# A Review on Traditionally Used African Medicinal Plant *Annickia chlorantha*, Its Phytochemistry, and Anticancer Potential

**DOI:** 10.3390/plants11172293

**Published:** 2022-09-02

**Authors:** Paromita Sarbadhikary, Blassan P. George

**Affiliations:** Laser Research Centre, Faculty of Health Sciences, University of Johannesburg, P.O. Box 17011, Johannesburg 2028, South Africa

**Keywords:** acetogenins, African traditional plants, *Annickia chlorantha*, cancer, drug discovery, medicinal plants, natural products, protoberberines, phytochemicals

## Abstract

*Annickia chlorantha* Setten & P.J.Maas belongs to the Annonaceae family and is a multi-purpose medicinal plant, which has been extensively used for the traditional treatment option for malaria in western and central Africa. Its phytochemical composition is dominated particularly by various biologically active protoberberines and acetogenins. This review aims to provide a comprehensive review on the traditional uses, phytochemical profiles, and the toxicology of this plant from a myriad of available publications. Even after its tremendous applications against several different human ailments, this plant has been underestimated for its anticancer potential. Herein, based on the phytochemical composition, we discuss the probable mode of mechanism for its antiproliferative activity, which highlights its importance for cytotoxicity screenings against cancer cells. Additionally, this article discusses several research questions and suggests the future directions of its applications in medicinal plant-based anticancer research.

## 1. Introduction

The increasing global burden of cancer incidence rate and death, as well as resistance development and adverse side effects associated with all the available anticancer therapeutic modalities, have become a matter of great concern [1,2,3,4]. These limitations have prioritized the quest for better anticancer drugs with more effectiveness and specificity and with fewer side effects [5]. Studies have shown that the natural substances have potential to impart selective toxicity against cancer cells along with being natural, they exert less side effects [6,7,8]. Age-long use of plants in traditional medicines is of great importance as remedies for several different human ailments [9,10,11]. Due to the lack of proper information, certain induced toxic effects and with the beginning of the era of “modern” drugs in the 19th century overtook the use of the direct crude extract of plants or plant parts [12,13]. The transformation of the pharmaceutical chemistry from a state of alchemy to an acknowledged branch of science started in 1805. For the first time, Friedrich Sertürner came up with the successful isolation of first pharmacologically active compound morphine from the opium plant [14]. Subsequently, numerous plant-based bioactive compounds have found their way in the treatment of several different diseases. As reviewed by Newman and Cragg, over a period of almost 39 years from 1981 to 2019, ~1881 drugs of all different categories of natural and synthetic entities have been approved by the US FDA (Food and Drug Administration) and different countries. Among these, 18.4% are biological macromolecules, 3.8% are unaltered natural product, 0.8% are natural product botanical (defined mixture), and 18.9% are natural product derivatives which have already been approved for all diseases worldwide. While concentrating on 247 total approved antitumor drugs, data show that 21%, 7.3%, 0.4%, and 17.4% are biological macromolecules, unaltered natural products, natural botanical products, and natural product derivatives, respectively [15]. This evidence proves the importance of plant-derived phytochemicals in the treatment of cancer. Further, the field of development of new synthetic anticancer drugs appears to be reaching its limits based on its chemical diversity and structure–activity relationship. On the other hand, plant derived natural products, offers the unique ness of chemical diversity resulting into diversity in their biological activities and properties [12]. All these have shifted the attention of researchers towards exploring plants in the search of effective and safer anticancer drugs [9,13].

Similar to other available traditional medicine worldwide, African traditional medicine based on plants is also one of the oldest and diversified therapeutic systems. Africa, a continent of enormous biodiversity resources, has been reported to be a land of ~40–60,000 plant species, with ~35,000 being endemic and among which ~5000 species have been reported to a medicinal potential due to their important secondary metabolites’ composition [16,17]. However, unfortunately, in spite of the huge potential and diversity, very few drugs from African plants have been commercialized globally, due to understudy and or under-exploration. Over the past few years, an increasing amount of scientific literature reporting the efficacy of understudied African medicinal plants has shown to represent an upward trend [18,19]. Further, Kuete and Efferth provided a review to discuss the potential anticancer efficacy of several African plants belonging to Asteraceae, Annonaceae, Amaryllidaceae, Acanthaceae, Apiaceae, Araliaceae, Compositae, Celastraceae, Caesalpiniaceae, Dioscoreaceae, Euphorbiaceae, Euphorbiaceae, Fabaceae, Iridaceae, Lauraceae, Moraceae, Mimosaceae, Olacaceae, Piperaceae, Poaceae, Rutaceae, Rosaceae and Zingiberaceae families against sensitive and resistant cancer cells. Most importantly, these plants or their derived products have traditional uses, but they have not been explored to treat cancers [17]. With all this rich background information, in this review, we try to provide an in-depth analysis of a multi-purpose and widely used tropical African medicinal plant *A. chlorantha* Setten & P.J.Maas which has been underestimated for its potential anticancer effectiveness [20].

## 2. Taxonomy, Biodistribution, and Botanic Description

*A. chlorantha* (Oliv.) Setten & P.J.Maas with a generic name *Enantia chlorantha* is a member of family Annonaceae and order Magnoliales. It is commonly known as African yellow wood, as well as also called by several different names in indigenous language Awopa, Osu pupa or Dokitaigbo (Yoruba), Osomolu (Ikale), Erumeru (Nigeria), Kakerim (Boki), Erenba-vbogo (Benin), Mfo (Boulou), Mpouley (Mabea), Njie (Douala), yellow moambi (English), and moambi jaune (French). *A. chlorantha* is an ornamental dense forest tree widely spread along Sub-Saharan Africa and distributed in the eastern and southern forest of Cameroon, southern part of Nigeria, Gabon, Guinea, Ivory Coast, Liberia, Angola (Cabinda), and DR Congo (Province Bas-Congo). This plant is primarily common in lowland rainforest, along roads and on slopes, at 150–850 m altitude [21,22].

This species is mainly represented by a small to medium-sized tree, which is usually straight and cylindrical, but sometimes fluted, and grows up to 25 m tall with bole branchless for up to 20 m and diameter up to 80–90 cm. The trees have smooth brownish gray to blackish outer bark, often with indistinct horizontal folds, while the inner bark is fibrous and bright yellow in color with a peppery-resinous smell. They grow with dense foliage and spreading triangular crowns, with tall and thin horizontal branches, curving down towards their tips. The color of the upper surface of their dry leaves is brown to gray-green, they have bifid or trifid of leaf hairs type pointing in all directions, sepal length of 8–12 mm in the latter, and elliptical petal shape with 0.6–2.0 cm length of the stipe [20,22].

## 3. Phytochemical Composition and Toxicity

The proximate component analysis of stem bark extract has shown to be high in crude fiber (72.25%) followed by 10.78% crude protein, 6.29% of carbohydrate content 3.78% of crude fat, with only 3.85% of moisture content and 2.48% of ash content [23]. While qualitative studies on phytochemical screening have shown the presence of phenolics, flavonoids, alkaloids, glycosides, reducing sugars, and saponins in both aqueous and ethanolic extracts of stem bark of *A. chlorantha*, the most used plant part with several reported medicinal properties [23,24,25], quantitative percentage was highest for alkaloids followed by phenols, sugars, saponins, and glycosides, and flavonoids being the least [23,24,25]. Phytochemical screening of ethanol crude extract of roots revealed the presence of alkaloids, reducing sugars, flavonoids, tannins, saponins, and glycosides [26]. Gill and Akinwuni, meanwhile, reported the presence of lignin and tannins apart from the reported alkaloids and saponins in bark and leaf extracts [27]. Among the alkaloids, *A. chlorantha* extracts are dominated by protoberberines which mainly include berberine, palmatine, 7,8-dihydro-8-hydroxypalmatine, jatrorrhizine, columbamine, and to a lesser extent canadine, pseudocolumbamine, phenanthrene alkaloids, and aporphines [20]. Further, the essential oil isolated from bark has been reported to contain ~20 volatile components, mostly the oxygenated sesquiterpenes which includes caryophyllene oxide, 1,5-epoxysalvial-4(14)-ene, humulene epoxide II, and spathulenol [28,29]. Extract from *A. chlorantha* dried stem or stem bark in 95% ethanol/water has been reported to be rich in acetogenins, a class of bioactive polyketides found exclusively in the Annonaceae family. Although, acetogenins have not been isolated from *A. chlorantha*, acetogenin-rich aqueous, hexane, and ethanol and methanol fractions of stem bark and stem have been shown to be effective for the treatment malaria [30,31].

Similar to any other herbal traditional medicine *A. chlorantha* also suffers from induced side effects and toxicity in animal studies. The leaf and bark decoctions *A. chlorantha* have shown possible acute and chronic toxicity in Swiss albino mice, whereby the extracts increased the number of nucleated cells in the spleen, liver, and peripheral blood [32]. Mice administrated with oral and sub-cutaneous doses of both the aqueous and ethanolic extracts reacted by itching leading to body scratching lasting about 10 min. Mean lethal dose (LD_50_) was recorded at doses of 0.7 g/kg and 43.65 g/kg for ethanolic and aqueous extracts, respectively. No fatality was recorded in mice administered with 0.2 g/kg subcutaneously and 20.0 g/kg orally of both the ethanolic and aqueous extracts while higher doses of both extracts resulted in deaths [33].

The toxicity studies carried out by Tan et al. revealed that aqueous stem bark extract at 1000 mg/kg upon oral administration resulted in histopathological changes in the liver, lungs, and kidneys, as well as increases in alanine transaminase, aspartate transaminase, and platelet count in Swiss albino mice. LD_50_ dose of >5000 mg/kg was obtained; however, medium-to-long term use at doses greater than 500 mg/kg can cause lung, hepatic, and kidney disorders [34]. Adebiyi and Abatan showed that although the oral administration of ethanolic extract of *A. chlorantha* stem bark was safer for albino rats at doses lower than 500 mg/kg body weight, at relatively high doses, it resulted in severe toxic effects such as decreases in the levels of packed cell volume, hemoglobin concentration, and red blood cell counts. Further, extract higher than 1000 mg/kg caused congestion in the heart and kidney of experimental rats [35]. Furthermore, the modified Ames assay showed an in vitro mutagenic effect induced by bark extract; however, this effect needs to be confirmed in vivo [36]. The studies conducted to investigate the possible reproductive and developmental toxicity by the stem bark aqueous extract on pregnant rats showed that less than 500 mg/kg dose did not induce any general visible toxic effects in dams and pups. However, doses more than 500 mg/kg caused persistent cystic glandular hyperplasia in the uteri, together with increased glandular epithelial cell proliferation [37]. Odoh et al. reported a LD_50_ of 4.325 g/kg in mice treated with ethanol extracts of roots [26].

Other than extracts, toxic effects of protoberberine alkaloidal fraction from stem bark ethanolic extracts were evaluated in mice injected intraperitoneally. A dose of 150 mg/kg was found to be relatively safe, while higher doses resulted in death. Lowers doses did not induce any pathological effects on the stomach, kidneys, esophagus, or liver, while it showed mild and moderate edema in the lungs [38]. However, acute toxicity studies in Swiss albino mice showed that oral administration of acetogenin-rich fractions and interface precipitates at a dose of 2 g/kg did not exhibit any evidence of in vivo toxicity [31].

## 4. Traditional Uses and Biological Activities

The *A. chlorantha* plant is endowed with multiple pharmacological properties such as analgesic, antioxidant, anticonvulsive, antidiabetic, anti-inflammatory, antimicrobial, antimycobacterial, antiplasmodial, antipyretic, antisickling, antitumor, antiulcer, antiviral, hepatoprotective, hemostatic, testiculoprotective, and uterus stimulation activities. Thus, traditionally, this plant’s parts, such as roots, stem, and bark, have been used in the treatment of several different human ailments such as anemia, bacterial infection, fever, infected wound, infective hepatitis, jaundice, leprosy spots, malaria, rickettsia fever, stomach aches, tuberculosis, typhoid fever, urinary tract infections, and yellow fever [20,21,39]. In traditional practice, decoction and extracts from stem bark of *A. chlorantha* have been mainly used to treat aches, boils, chills, fever, hepatitis, malaria symptoms, sore, spleen in children, vomiting, wounds, and yellow bitter either alone or in combination with stem bark extracts of other plants such as *Rauvolfia vomitaria* and *Fagara macrophylla* and/or *Nauclea latifolia*. Further, oral administration of stem bark decoction has been reported to be effective against hepatitis, intestinal worms, intestinal spasms, jaundice, sexual asthenia, typhoid fever, and urinary tract infections. Moreover, stem bark has been shown to be effective as hemostatic agent and as uterine stimulant as well as treating leprosy spots, stomach problems, and some forms of skin, gastric, and duodenal ulcers. Dried stem bark has also been used to treat hepatic disorders, malaria, tuberculosis, and ulcers, while infusion of bark showed effectiveness for the treatment of cough and wounds. Its root decoction has been used for its antimalarial, anti-jaundice, and antipyretic properties [40].

With advancement in modern medicine, the biological activities of traditionally used *A. chlorantha* extracts and isolated phytochemicals have been validated with scientific evidence, which is summarized in Table 1.

## 5. Anti-Tumor Effects of *A. chlorantha* Extracts

Methanolic leaf extracts from *A. chlorantha* have been shown to exert its antiproliferative cytotoxic effects against two breast (BT20 and MCF-7) and two colorectal cancer cells (SW480 and SW620) when treated for 24 h. Treatment with 0.96 μg/μL inhibited the cell viability to 80%, 93%, 80%, and 96% in BT20, MCF7, SW480, and SW620, respectively [68]. Another study also reported the anticancer property of chloroform and ethyl acetate isolates of bark extracts of *A. chlorantha* against human prostate cancer cell lines PC-3 and MCF-7 breast cancer cell lines. Both the isolates showed potent cytotoxicity towards both the cell lines. Chloroform and ethyl acetate isolate showed a cytotoxic concentration 50 (CC_50_) of 3.84 CC_50_/mL and 4.87 CC_50_/mL in MCF-7, respectively, while CC_50_ of the both the isolates were >10 CC_50_/mL for PC-3 [69]. Methanol extracts of *A. chlorantha* bark extract showed selective activities against human mesothelioma cell line SPC212 and hepatocarcinoma cells HepG2 with IC_50_ of >1.59 μg/mL and >12.31 μg/mL, respectively, while no toxicity was reported for lung cancer (A549), colorectal adenocarcinoma (DLD-1), or breast cancer (MCF-7) cell lines [70]. 

## 6. Possible Anticancer Effects

### 6.1. Protoberberines

As presented by the above-mentioned studies, this plant has seriously been underestimated, thus no reports of thorough phytochemical analysis for its antineoplastic potential were found. However, extract of *A. chlorantha* has been shown to contain high content of protoberberines, an isoquinoline alkaloid [39,71]. Thus, antiproliferative activity of protoberberines can be a possible mode of action to *A. chlorantha* extract-induced cytotoxicity against cancer cells. Few reports have shown protoberberine alkaloid extracts from different protoberberines such as berberine, palmatine, columbamine, and jatrorrhizine [39,64].

**Figure 1:**
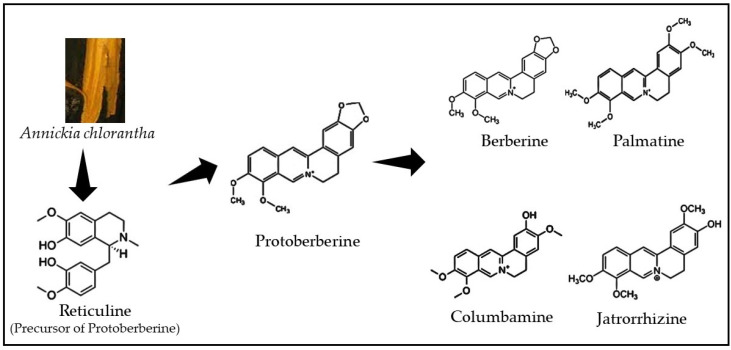
Chemical structures of protoberberine alkaloids class.

Isoquinoline alkaloids form the diverse group of phytochemicals of natural products kingdom and offer a promising chemical platform for discovering new chemotherapeutic drugs. Chemically, they possess an isoquinoline moiety, a heterocyclic compound consisting of a benzene and pyridine ring fused at C3/C4 of the pyridine ring (Figure 1). Structurally, isoquinoline alkaloids are classified into the subgroups of aporphine, benzylisoquinoline, benzo[c]phenanthridine, emetine, morphine, protoberberine, protopine, phthalide isoquinoline, and pavine, whereas isoquinoline alkaloids berberine, palmatine, columbamine, and jatrorrhizine found in *A. chlorantha* extracts belongs to the protoberberine class [39,72]. 

**Figure 2:**
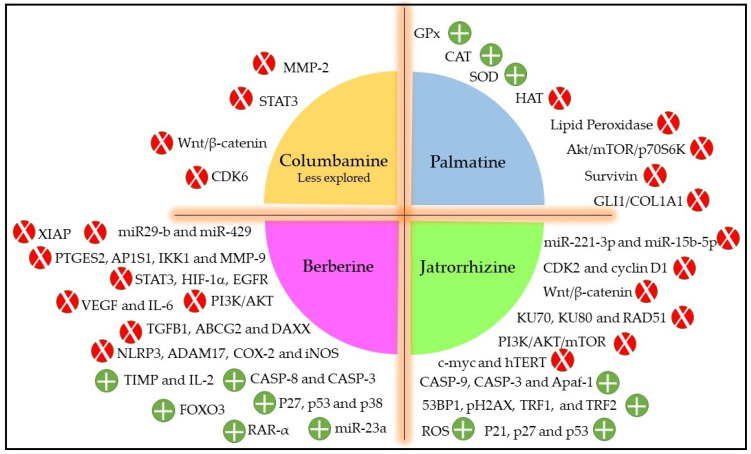
The probable antitumor mechanism of different protoberberines presents in *A. chlorantha* extracts. Red color indicates inhibition/reduction and green color indicates increase/promotion.

In general, protoberberines inhibit cancer growth and progression via several different mechanisms which include cell cycle arrest, inducing cell death by apoptosis and autophagy, inhibiting cell proliferation and invasion, as well as regulating the expression of microRNA, telomerase activity, and tumor microenvironment, which usually varies for different cancer types [73,74]. Due to its structure, protoberberines intercalate strong with the DNA helix structure, which results in topoisomerase-I and -II poisoning resulting in cancer cell antiproliferation [75]. Protoberberines also exhibit cell cycle arrest by downregulating Cyclin D1 and upregulating CDK inhibitors such as p21 and p27 [76]. Most importantly, berberine induces cell death by apoptotic pathway by increasing the expression of caspase genes *CASP3*, *CASP8*, and *CASP9* and proapoptotic genes *BAK1*, *BAX*, and *BIK* with simultaneous suppression of antiapoptotic genes *BCL2*, *BCL2L2*, *BNIP1*, and *BNIP3* expressions [77]. Protoberberines also exert their anticancer potential by modulating several different mitogen-activated protein kinases signaling pathways, such as ERK1/2, p38 MAPK, and JNK pathways. Berberine has also been shown to inhibit the important transcription factor AP-1, which play a key role in proliferation, inflammation, and apoptosis, as well as being involved in the inhibition of N-acetyltransferase activity and COX-2 transcription [78]. Protoberberines also act as antimetastatic agents by reducing the levels of phosphorylated forms of JAK2 and STAT3, which decreases the expression of matrix metalloproteinases (MMP-2 and MMP-9) due to interrupted COX2/JAK/STAT signaling. Further, protoberberines also prevent angiogenesis progression by inhibiting transcription factors such as NF-κB and HIF-1 and PI-3K/AKT pathway which subsequently reduce the expression of angiogenesis-promoting factors, i.e., COX-2, HIF, VEGF, and IL-8 [73,79]. Protoberberines have also been shown to target the AMPK/mTOR/ULK1 pathway resulting in the activation of autophagy cell death. Berberine also causes alteration in the inflammatory tumor microenvironment by downregulating the caspase-1/IL-1β signaling pathway, which results in inhibition of cancer cell proliferation and invasion [20,73,80]. In Figure 2, we compiled different possible modes of action of *A. chlorantha* extracts based on the reported cytotoxic activities induced by each type of protoberberine [73,81,82,83,84,85,86,87].

### 6.2. Acetogenins

**Figure 3:**
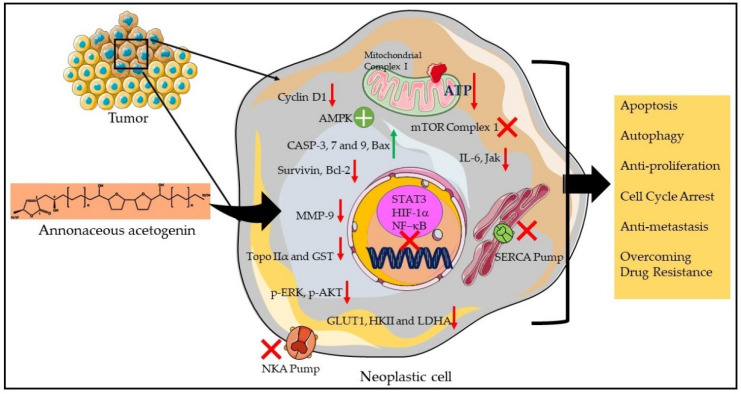
The probable acetogenins-rich *A. chlorantha* extracts induced antitumor mechanism. Red arrow indicates inhibition/reduction, green arrow indicates increase/promotion, green circle represents activation, and red cross indicates inhibition of proteins/complexes.

Structurally, the Annonaceous acetogenins are characterized by a long aliphatic chain with a terminal methyl-substituted α,β-unsaturated γ-lactone ring with one, two, or three tetrahydrofuran (THF) rings located along the hydrocarbon chain together with a number of oxygenated moieties such as hydroxyls, acetoxyls, ketones, and epoxides and/or double and/or triple bonds. Mechanistically, acetogenins are reported to exhibit a broad range of biological activities such as antimicrobial, antiparasitic, antitumoral, cytotoxic, immunosuppressive, and pesticidal effects. To date, more than 500 Annonaceous acetogenins have been described; however, unfortunately, no acetogenins have been isolated and characterized from the fractions of *A. chlorantha* extracts. Nonetheless, acetogenins from other Annonaceous species have been shown to induce strong antineoplastic efficacy against different human cancer both in vitro and in vivo, as well as reported to overcome chemodrug resistance in multidrug resistant (MDR) tumors [88,89,90,91]. Thus, presence of high amount of acetogenins in *A. chlorantha* extracts can be another mechanistically possible reason contributing to its anticancer potential. Based on the available literature, Figure 3 represents the different acetogenins -mediated anti-tumor effects. 

Similar to protoberberines, acetogenins also exert their anticancer efficacy by several different mechanisms. The most prominent biologic activity is its ability to inhibit the mitochondrial Complex I, leading to blockade of oxidative phosphorylation. This subsequently results in a decrease in ATP which finally inhibits pathways inducing cell death, or cell cycle arrest. Acetogenins have been reported to promote apoptosis by upregulating the activity of *CASP3*, *CASP8*, and Bax pathways while downregulating the expression of surviving and Bcl-2, thereby enhancing apoptosis. They also induce autophagic cell death by targeting the AMPK/mTOR pathway. Acetogenins inhibit cyclin D1 expression leading to G1/S phase cell cycle. Antiproliferative activity of acetogenins is also exerted by reducing the expression of HIF-1α and NF-κB and decreasing the protein levels expressions of the glucose transporter GLUT1 and the HKII and LDHA enzymes. Interestingly, acetogenins promote cell death by the inhibition of the NKA and SERCA pumps. Importantly, acetogenins have been shown to overcome chemodrug resistance in cancer by downregulating the expression of the drug-resistant genes *MDR1* and *MRP1* as well as topoisomerase IIα and glutathione S-transferase. Acetogenins reduce metastasis in tumor xenografts by reducing the levels of the MMP-9, and downregulating IL-6, Jak, and various phosphorylated activators of the STAT3 pathway [90,91].

## 7. Conclusions

In summary, *A. chlorantha* has been proven to be a promising traditional medicine for the treatment of protozoal infections and has been studied extensively in terms of taxonomy, biological properties, and conservation. However, this plant has been understudied with respect to its extensive phytochemical composition, which could a probable reason for underestimating the investigation of it being an antitumor drug. In general, the therapeutic potential of chemically complex plant extracts depends on and varies with the interactions among compounds and their proportions within the extract. Thus, extracts or more importantly isolated phytocompounds of this plant should be considered for cytotoxicity screenings against cancer cells to explore the possibility of its application in cancer treatment. The extracts of this plant are rich in protoberberines and acetogenins, which have a proven record of strong antiproliferative activity against cancer cells both in vitro and in vivo. Although several protoberberines have been isolated from their extracts, none of them have been tested against cancer. Adding to that, unfortunately, none of its acetogenins have been isolated and identified, which are well-known anticancer compounds. However, in vivo bioavailability and administered doses of the phytochemicals such as protoberberines and acetogenins have always been a concern due to their hydrophobicity issue, which makes them insoluble in aqueous biological solvents. Thus, in this regard, nanotechnologically modified drugs and drug-delivery systems offer a possible solution of clinical applications of potent phytochemical with better cancer treatment outcomes [92,93]. Further in vivo acute and sub-chronic toxicity analysis in mice suggest precautionary use. Therefore, their clinical applications warrant careful and systemic human studies to better understand their pharmacodynamics and pharmacokinetics. Furthermore, this plant is already being threatened with extinction, due to its traditional medicinal uses, over-harvesting, and destruction. Thus, this review is an effort to provide a thoughtful discussion on the importance of *A. chlorantha* for investigating its antitumor activity which could represent a major part in both traditional and modern healthcare systems as well as in future cancer research.

## Figures and Tables

**Table 1:** Biological activities of extracts and compounds isolated from *A. chlorantha*.

Biological Activities	Plant Part/Sample/Isolated Phytochemical Used
**Antiamoebic**	Isolated Protoberberine alkaloids [41]
**Antibacterial**	Aqueous extract of stem bark [42,43] Isolated essential oils [29] Ethanol extract of root [26] Ethanol extracts of stem bark and stem and methanol fractions [44] Alkaloidal extracts of stem bark [45] Jartrorrhizine-1, canadine-1, argentine, jartrorrhizine and berberine (molecular docking analysis) [46]
**Antifungal**	Isolated essential oils [29] Ethanol extract of root [26] Alkaloidal extracts of stem bark [45] Argentinine-1, columbamine-1, jartrorrhizine-1, pseudocolumbamine-1 (molecular docking analysis) [46]
**Anti-inflammatory and antioxidant properties**	Boiled water bark extract [47] Isolated essential oils [29] Methanol-dichloromethane bark extracts [48] Methanol, n-hexane, chloroform, ethyl acetate and aqueous fractions of stem bark extracts [25]
**Anti-leishmania**	Aqueous extract stem bark [49] Isolated protoberberine alkaloids [50,51]
**Antimalarial**	Aqueous and ethanol extract of stem bark [49,52] Leaf and bark decoctions [32] Boiled water bark extracts [53,54] Methanol extract of stem bark [55] Aqueous, hexane, ethanol and methanol (acetogenin-rich) fractions of stem bark and stem [30,31] Isolated protoberberine alkaloids [41,56] 1,3-dibenzoyl-2-azepanone and 3,5-bis(1,1- dimethylethyl)-phenol (molecular docking analysis) [57]
**Antipyretic properties**	Aqueous extract of bark [58] Aqueous and ethanolic extracts of stem bark [24]
**Anti-trypanosoma**	Ethanol extract of root [26] Aqueous extract of stem bark [49] Isolated protoberberine alkaloids [51,59]
**Gastroprotective**	Ethanol extract of stem bark [60] Isolated Protoberberine alkaloids [61,62]
**Haematological**	Ethanol extract of stem bark [35]
**Hepatoprotective**	Hepasor (a protoberberine-containing extract) [63,64] Hexane, chloroform, ethyl acetate and methanol extracts of stem bark [65]
**Testiculoprotective**	Aqueous extract of stem bark [66,67]

## Data Availability

Not applicable.
